# Experiences of medical students and nursing trainees from unexpected death through simulation training

**DOI:** 10.1186/s12909-023-04638-x

**Published:** 2023-09-14

**Authors:** Dominik Hinzmann, Marjo Wijnen-Meijer, Laura Corazza, Veronika Becker, Simone Kagerbauer, Rainer Haseneder, Pascal O. Berberat, Nana Jedlicska

**Affiliations:** 1 TUM Medical Education Center, TUM School of Medicine, Technical University of Munich, Munich, Germany; 2 Department of Anesthesiology and Intensive Care, TUM School of Medicine, Technical University of Munich, Munich, Germany; 3 Department of Anesthesiology and Intensive Care Medicine, University of Ulm, Ulm, Germany

**Keywords:** Simulation training, Death simulation, Debriefing, Advance directive

## Abstract

**Background:**

Dying in simulation training is controversially discussed. On the one hand, the danger of an emotional overload of the learners is pointed out. On the other hand, dying in simulation settings is addressed as an opportunity to prepare future health professionals to deal with patient death. The present study investigates how medical students and nursing trainees experience the sudden death of a simulated patient and how and under which conditions it can be valuable to simulate the patient’s death.

**Methods:**

At the TUM School of Medicine in Munich, Germany, we developed an interprofessional, simulation-based course in which participants were unexpectedly confronted with a cardiac arrest scenario within which resuscitation had to be discontinued due to an advanced directive. After the course, focus groups were conducted with nine medical students and six nursing trainees. Data were analysed using Grounded Theory techniques.

**Results:**

The participants reported low to high emotional involvement. The active renunciation of life-sustaining measures was felt to be particularly formative and caused a strange feeling and helplessness. Questions of what could have been done differently determined interviewees’ thoughts. The participants appreciated the opportunity to experience what it feels like to lose a patient. The course experience encouraged interviewees to reflect on dying and the interviewees explained that they feel better prepared to face death after the course. The unexpected character of the confrontation, presence of the advanced directive and debriefing positively affected the impact of the simulation.

**Conclusions:**

The study recognises simulation training as a promising approach for preparing future health care professionals to encounter a patient’s death.

**Supplementary Information:**

The online version contains supplementary material available at 10.1186/s12909-023-04638-x.

## Background

The main focus of the medical school curriculum is on sustaining life and cure, but any cure is temporary and death and dying are natural events [1–3]. Reports about medical education of end-of-life-care are divided: while some students are regularly confronted with these subjects [4], others report the opposite, and a lack of (psychological) support [2, 3, 5, 6]. One way to train students to deal with death in critical and life-threatening moments is simulation training, which is a way to bridge the gap between classroom and clinical practice and enable students to learn in safe environments [3]. During simulated social situations, students respond in the same way and slip into their assigned roles as in real situations [7]. That makes simulation training an effective way [8] to teach skills needed in life-threatening patient care situations [7], such as resuscitation. In these simulations, manikins are used as patients and students are actively involved [8].

### Simulation of death

Although the demand for education about death is great, there is a considerable controversy about dying in simulation training [3]. There is no clear recommendation as to whether the simulation of a patient’s death, and therefore the actions to be taken when faced with a cardiac arrest and resuscitation, should be part of medical education and, if so, at what stage. For example, Bruppacher et al. suggests to train simulated death only with senior trainees and warns about high emotional tension and stress that might occur in simulations that comprise patients death [9]. DeMaria et al. summarised that learners experience stress during death simulation training, but without negative outcomes for the students [10]. Other authors argue that death of patients is the most common outcome in resuscitation of real patients with cardiac arrest and these emergencies occur to practitioners of all experience levels [11]. They outline that students have to deal with death in the clinical environment and therefore they need to be prepared to respond appropriately to death in every stage of education. They highly recommend to carry out these simulation trainings during medical school [5, 6, 11–13]. A great advantage of simulation training is that students can witness and learn from the results of their actions without harming patients’ lives [14]. Thereby, the experience can decrease fear, anxiety, and feelings of inadequacy in students [12, 14].

When conducting a simulation training with occurring death, several considerations must be made, such as predefined learning objects, ethical aspects, individual attitude towards death, required skills of the trainer and setting. Predefined learning objects means that students get prepared for every possible situation which may occur during the simulation training. In the literature, it is discussed if and how the simulation of death as an unpredefined learning object affects the student’s learning process and emotions [3, 9, 14]. According to Weiss et al. the perceived self-efficacy – a person’s perception of being able to accomplish a task – is not affected by whether medical students are warned or not of the possible death of a manikin [13]. But, the simulation of death can be very demanding on the psyche of medical students, hence instructors must consider religious, ethical and psychological aspects in advance and during simulation [3, 7, 9, 15]. Therefore, patient death simulation and the actions to be taken in the event of cardiac arrest should be an explicit learning object rather than used to heighten emotional tension and stress [9].

Leighton & Dubas described three types of patient death during simulation training: (a) expected, (b) unexpected death, and (c) death resulting from action or inaction [14]. In an (a) expected death scenario learners are warned in advance of the possible death (e.g. through prebriefing). While students in an (b) unexpected death simulation are unprepared, there the death is purposefully incorporated by the instructor. This distinguishes from (c) death resulting from action (e.g. death following a medication overdose) or inaction of students (e.g. death due to failure to recognise a worsening condition). In that case, death is a consequence of inappropriate nursing action or failure to provide appropriate care in a timely manner and is unexpected for students and instructors [14]. For students, unexpected death leads to more distress and psychological trauma than expected death [1].

Additionally, to these three types of patient death that can be simulated in a laboratory, there are cases where physicians have to act according to the last will of patients. End-of-life decisions involve considerations of preferences and priorities regarding life-prolonging and life-limiting interventions, palliative treatment and preferred care/death settings [16]. A legally binding Advance Directive (AD) documents the wishes of patients in the event that they no longer have decision-making-capacity [17].

The experience of death simulation has the potential to be stimulating and empowering for students and increase their resilience [3, 8, 12], but without highly skilled and experienced trainers it can also be stressful or shameful [8]. Educators need to be fully prepared to recognise and address psychological distress that scenarios may invoke [8]. Especially, the individual way people deal with death must be considered: for example, people may feel discomfort or fear when confronted with death, others accept it as normal, or as God will. Additionally, some see it as failure of the medical team, which arises when medicine is seen solely as life-sustaining [5, 18].

In general, it is important to be aware that the simulation of death, and therefore the actions taken when faced with an AD during resuscitation of a cardiac arrest, can be appropriate in some situations, but it might be unwise in others when it puts students under too much stress [7]. Therefore, the conditions under which the simulation of death is valuable for students must be considered.

### Prebriefing and debriefing

In the literature, prebriefing and debriefing are recommended to support the simulation training [7–9, 19–21]. Prebriefing is held beforehand and may lead to expected death simulations, while debriefing sessions are held after a simulation training and reflect the experience [8]. The topics of the prebriefing are: the purpose of the simulation, the learning objectives, the students’ expectations, a review of simulator features, the possibility of death and other risks, and an explanation of the debriefing [7, 8, 22]. In the prebriefing, the students get to know what is expected of them in the simulation and what basic rules they have to follow [22]. Truog & Meyer consider prebriefing important for early learners [8], to minimise psychological distress and manage expectations of students [7, 8]. It might be left out during a realistic clinical simulation for advanced students [7]. An advance warning may lead to biased results, as the simulation may not replicate reality afterwards and could prepare students for the emotional shock they will experience [7].

Feedback, including debriefing, is often referred to as the most important component of simulation-based medical education [21]. Debriefing is defined by the American Psychological Association as “an intervention immediately following a traumatic event that aims to mitigate long-term distress” [23]. During this process significant learning occurs [22].

Lederman identified three phases of the debriefing process: the aim to review the experiences that the students had, evaluating the impact of the simulation, and the meaning attributed to the students [24]. In the first phase – systematic reflection and analysis – the participants are introduced to a systematic self-reflective process about the experience they have had previously. In the second phase – intensification and personalisation – the students reflect their own individual experiences and the meanings for them. During the last phase – generalisation and application – the students use their own individual experience to the broader application and impact of the experience [24].

Cheng et al. and Bruppacher et al. identified that structured debriefing has positive effects on the improvement of learners’ critical thinking and clinical judgement [9, 19].

### Aim of study

The present study describes and evaluates an interprofessional simulation-based emergency management course in which the simulated patient dies according to his AD.

The research questions are:

(1) How is the death of a simulated patient in an emergency management course perceived and experienced by medical students and nursing trainees?

(2) How and under what conditions can it be valuable to simulate the death of a patient?

## Methods

### Educational intervention

#### Course description

We developed an interprofessional, simulation-based course focusing on teamwork and communication skills for everyday clinical and emergency situations (“Training Interprofessional Teams for Daily Clinical Practice and Emergencies”). The course participants included medical students from seventh to ninth semester of the medical school and third year nursing trainees of the nursing school. Medical students were invited to participate in the course on a voluntary basis, while for nursing trainees participation was compulsory. The course consisted of five sessions with a duration of 3 to 3.5 h per session. The five sessions were distributed over two and a half weeks in December 2019. The course took place in the Medical Training Center of the Technical University of Munich (TUM). Each session was supervised by three to five trainers with medical and nursing backgrounds and experiences in debriefing.

### The fourth session: Acute Care problems II – when patient dies

The goal of the fourth session was to train the course participants for emergency situations and to raise their awareness that resuscitations are not always successful and that this experience is also part of the daily clinical practice. The aim was to promote ideas for self-care after experiencing a patient’s death. Learners should be encouraged to reflect on their own professional role in dealing with dying and death. To achieve the defined objectives, the participants were confronted with a simulation in which the manikin could not be resuscitated according to their AD. The AD was integrated to investigate how the presence of an AD influences the participants’ experience and perception of the simulation. The participants did not receive any education on AD and the consequences of them prior to the simulation in the course. The knowledge among participants around AD was also not recorded. Mention of AD was deliberately avoided in order not to arouse suspicion among participants about the outcome of the scenario. In general, the topic of patient death and living will, as well as how to deal with dying patients and their families, is covered in detail in nursing education. As part of medical studies, these theoretical backgrounds and legal frameworks are addressed in an obligatory cross-sectional subject of palliative medicine. In both teaching concepts, however, the personal handling of the subject of a patient’s death and the potential burden that results from it are not discussed and are not given any space. After all, this was one of the reasons for addressing death in the context of a protected learning environment such as the simulation. The session consisted of three phases:

#### 1. Prebriefing

During the prebriefing, the trainers welcomed the participants and ensured a pleasant atmosphere to promote learning and limit participants’ stress. They then presented the general training objectives of the session without mentioning the possibility that the manikin might die. Therefore, the title of the fourth session (“Acute Care Problems II – When patient dies”) was not made public in advance. It was a conscious decision not to warn participants about the possible death of the manikin. They should experience the unexpected death of the patient as authentically as possible. The prebriefing took about 15 min.

#### 2. Simulation intervention

The prebriefing was followed by the simulation. Two medical students and two nursing trainees who volunteered participated actively in the simulation. All other course participants observed the action on the screen in the debriefing room. In preparation for the simulation, the roles were distributed and the scenario participants were given a brief description of the scenario situation. The simulators and the material were familiar to the participants from the previous sessions. The scenario situation involved a 65-year-old patient with metastatic colon cancer who is found lifeless in the patient’s room by scenario participants. The scenario participants who embodied the medical and nursing staff, start resuscitating the patient. After about five minutes of unsuccessful resuscitation, a nurse, embodied by a trainer, enters the room, and brings the AD of the patient. The AD, in which the patient explicitly wishes to forgo life-saving measures, is handed over to the scenario participants. It was then left up to the participants themselves how they wanted to deal with the AD. In fact, one scenario participant read the AD, whereupon the participants stopped resuscitation. The simulation situation was then ended by the trainer’s announcement.

The patient was simulated by a high-fidelity manikin (Resusci Anne Simulator). The simulation lasted 30 min in total: 15 min preparations (technical preparation, scenario description and role distribution) and 15 min the simulation itself. The simulation was audio and video recorded.

#### 3. Debriefing

Immediately after the simulation, the debriefing took place with the entire group. Following Lederman’s three-stage debriefing model [24], at the beginning of the debriefing, all participants were invited to express their spontaneous feelings and thoughts about the simulation without inhibitions. In this way, the trainers responded to the participants’ statements and conveyed an understanding of their reactions. In the second phase of intensification and personalisation, the key moments of the simulation were watched and discussed based on the video recording. With this regard, the trainers addressed practical issues and discussed how to handle an emergency situation. In the third phase of generalisation and application of the experience, the conversation focused on the discussion of the patient’s death. The trainers emphasised the individual character of the perception of such experiences. The strategies for dealing with the death of a patient were also addressed. Throughout the debriefing, the trainers made sure that participants had enough opportunity to make comments and ask all their questions. The session was then concluded with a final feedback round in which participants were asked to share their general impression of the session in one sentence. The debriefing and final round lasted a total of 2 h. The unusually long duration of the debriefing is due to the challenging topic. We wanted to ensure that we had enough time to discuss the topic comprehensively and to thoroughly address the questions and thoughts of the participants.

The fourth session was held by four trainers who operated the manikin and guided the prebriefing and debriefing. The professional background of trainers was specialist in anaesthesiology (n = 2), intensive and anaesthesia care nurse (n = 1) and anaesthesia technician (n = 1). All trainers had several years of experience in simulation team training and debriefing. One anaesthesiologist had special training in trauma management and peer support.

### Ethical approval

for the educational intervention and for the study was obtained from the ethics committee of the Technical University of Munich School (registration number: 361/17 S). The present study is guided by the research ethics principles of the Working Group of Medical Ethics Committees in Germany. In accordance with the principles, efforts were made to ensure no harm, a favourable balance of benefits and risks, and respect for the study participants. Psychosocial support was also offered to the participants in the session itself, so that it was possible to counteract potential group pressure or other dynamics without deliberately exposing individuals to the group. However, this offer was not taken up. Written informed consent for involvement in the study was obtained from all participants before starting the data collection in the fifth session.

### Data collection

We chose a qualitative approach to examine the impact of the death of a simulated patient on medical students and nursing trainees. The qualitative approach was considered appropriate as its exploratory and flexible nature allows to explore previously unknown phenomena, broaden the scientific perspective and uncover themes that initially may not have been considered by the researchers. The open nature of the qualitative approach enables to reveal interviewees subjective perspectives and gives a detailed understanding of the phenomenon.

We decided to conduct focus group interviews to collect the data. It is crucial for the focus groups that all interviewees have experienced a certain situation. The conversation is then focused on illuminating how the situation was subjectively perceived, what of it was perceived and how [26]. Focus groups were considered appropriate as the dynamic nature of the group discussion can stimulate the spontaneous ideas that might otherwise remain hidden. Moreover, an interactive and supportive group environment can promote discussion of sensitive topics. As participants exchange opinions, they consider their own views in comparison to those of others, which can encourage participants to refine their thoughts, and extensive data can be collected [27]. The danger of one person dominating the conversation was minimized by the interviewers, who had ample experience in moderation.

All participants who attended the fourth session were invited to participate in focus group interviews. For the interviews, the participants were divided into three subgroups: Medical students (n = 4) and nursing trainees (n = 7) constituted the first and second subgroup. The third subgroup consisted of the scenario participants (n = 4). We decided to conduct the focus group interviews within each profession to create a safe atmosphere for the interviewees to freely express their opinions. Since we assumed that the scenario participants would experience the simulation situation more intensively, we decided to interview scenario participants in a separate focus group. The three focus group interviews were conducted simultaneously at the beginning of the fifth session, one week after the simulation of CPR termination (fourth session). The focus groups were each facilitated by a single researcher (VB, MWM or NJ). No other individuals were present. None of the researchers was involved in the teaching session in order to allow the interviewees to speak freely. All researchers had prior experience in moderating interviews. The interview guideline included open-ended questions to elicit participants’ perceptions and reactions to the death of the simulated patient. The guideline sought to capture the interviewees’ thoughts and feelings in response to the death of simulated patient, and the particularly defining, and challenging moments of the scenario. The guideline also explored interviewees’ engagement with the simulation experience (scenario situation and the debriefing) after the session, and the extent to which participants found the experience valuable. The discussion of how the topic was handled during the session concluded the interview. The open-ended strategy to the interview questions was intended to elicit interviewees’ experiences without influencing the manner in which their stories would be told. Additional file 1 shows the interview guideline in detail. After the focus groups, participants were asked to complete a short questionnaire for the recording of sociodemographic data. The interviews lasted between 40 and 80 min (an average of 58 min). The focus groups were audio taped and subsequently transcribed verbatim by a professional transcription service.

### Data analysis

Although our study is not to be understood as a classic Grounded Theory (GT) study, we used techniques of GT to analyse the data. The theoretical coding, which is the core of data analysis in GT, is the process of dividing data into sections of meaning and assigning codes to them. However, these are not descriptive and content-reducing, but aim at the development of a theory. In the process of coding the different codes or concepts are abstracted more and more, and their theoretical content is condensed. We chose coding techniques of GT because it allows for an open attitude towards the nature and content of the findings while still providing a systematic approach to data analysis [28]. In GT, which aims to develop theory inductively and iteratively from the data itself, coding consists of three steps: open, axial, and selective coding [26, 29]. In the first step of open coding, we extensively examined our data, ‘broke down’ data analytically, identified relevant concepts and grouped similar concepts together into categories. The concepts here are constructs generated from the data. They are not a mere summary of the text but describe how the text relates to a particular phenomenon. Categories are conceptualised codes in which the interrelationships of different codes become visible. Codes itself are units of meaning named by single words or keywords, often initially close to what is said. We completed line-by-line coding to ensure that the analysis remained close to the data and interviewees’ perspectives. Moreover, line-by-line coding fostered to remain open to the data, to detect otherwise undetected patterns, and thus, to reduce the risk of missing important themes in the analysis [28]. In the second step of axial coding, the categories developed during open coding were further elaborated, related to each other, and their relationship to each other was explored. In this context, in line with the interactive nature of GT, the preliminary concepts developed in the first step of open coding were revised and additional concepts were developed. As a result of the axial coding, several core categories emerged that represent the central thesis of the study. In the third step of selective coding, all categories were unified around core categories, and categories that needed further explication were filled-in with descriptive details. The coding process was limited to concepts and categories that had a sufficiently significant relationship to the core category and were thus relevant to theory building. At each level of analytical work, we used a technique of constant comparison: the sections of data were compared with other data sections, the concepts and categories developed were constantly compared with other concepts and categories for similarities and differences. This constant comparison helped to refine and elaborate the concepts and categories found, but also to explore the field in its diversity. Moreover, the constant comparison helped to sharpen, develop and revise our theoretical assumptions [26]. We used MAXQDA 22 for the data analysis.

## Results

We identified two main themes with regard to medical students’ and nursing trainees’ experience of the death of a simulated patient in an emergency management course. The first main theme *perception of the death of the simulated patient* captures the emotional and cognitive experience of the situation. The second main theme *value of the experience* captures the value of the simulation and the characteristics that significantly determine the success of the simulation. After a brief presentation of the demographic characteristics of the participants, these themes will be discussed in detail below.

### Demographic data

A total of 9 nursing trainees and 6 medical students participated in the focus groups. Of 15 participants, 13 were female. The participants ranged from 19 to 27 years of age, with a median age of 23. Table 1 shows the demographic characteristics of the participants.

**Table 1:** Demographic characteristics of the participants

	Medical students N = 6	Nursing trainees N = 9	Total sample N = 15
Gender (%)			
Female	4 (67%)	8 (89%)	12 (80%)
Male	2 (33%)	1 (11%)	3 (20%)
Age in years, mean (range)	23 (22–25)	23 (19–27)	23 (19–27)
Year of training	4. to 5. year	3. year	-

### Perception of the death of the simulated patient

#### Emotional reactions

Various interviewees described strong emotional consternation in response to termination of CPR. The novelty of the experience was named as the cause for being strongly affected.

Our interviewees described various emotions in reaction to the unexpected death of the simulated patient. The appearance of the AD initially made the interviewees feel deceived, since the outcome of the scenario was already predetermined and interviewees’ efforts to save the patient were aimless. At first, the interviewees felt they had been lured into an ‘ingenious trap’ and were initially unable to grasp the scope and significance of the living will.

Furthermore, the interviewees described feeling strange in response to the appearance of the living will.

“It was simply, it felt strange. Not right, but somehow also not wrong. Just very weird.”^1^ (Scenario participant)

The decision not to take life-sustaining measures despite the available treatment options was felt to be particularly strange and inconvenient. Actively foregoing life-saving measures and allowing death to occur was perceived as contrary to the nature of medical and nursing practice and custom.

Moreover, the discrepancy between the long duration of resuscitation and abrupt cessation of life-saving measures after reading the AD evoked the strange feeling. Lack of experience with death and inadequacy with dealing with death were mentioned as causes of the strange feeling. The end of the scenario, which was perceived as abrupt, was also found oppressive by some interviewees.

“Okay, yes, advance directive, okay. It says that he doesn’t want to continue living, so, yes. Yes, but that’s not possible (laughs). You have to resuscitate him and it’s not right that you wouldn’t try something just because of that.” (Nursing trainee).

Interviewees moreover addressed the uncertainty triggered by the unexpected appearance of the living will. Uncertainty applied to the meaning of the living will for one’s own actions on the part of the medical team. The questions of whether one may stop resuscitating or even whether one must stop resuscitating dominated interviewees’ thoughts. The finality and irreversibility of the consequences of the decision to comply with the patient’s will and discontinue life-saving measures was stressed as a factor that made it difficult for several interviewees to accept and comply the AD. Furthermore, there was uncertainty regarding the accuracy or validity of the AD. The team was concerned whether the AD was signed and whether it was signed by the patient.

With this regard, the interviewees also addressed their ambivalent attitude towards the AD. In particular, the uncertainties about whether the patients had received detailed information when the living will was drawn up and whether patients, as lay people, were aware of the scope of their decision made the interviewees doubt the AD. The reaction described by the interviewees that the living will was misinterpreted, the impulsive response of the interviewees wanting to ignore the patient’s wish to forgo life-saving measures, and the hope that there might be a turnaround in the situation illustrate the burdensome nature of the living will.

The interviewees also expressed their bewilderment, due to the discrepancy between the active efforts to save the patient and the passive watching in the next moment as a reaction to the reading out of the AD.

“Yes, so you’re somehow stunned, that you can’t just/ you know/ have never experienced it like this, that you just don’t do anything anymore and stop.” (Scenario participant).

Various interviewees described feeling helpless in the situation. On the one hand, not taking life-saving measures made the interviewees feel helpless. On the other hand, helplessness was evoked by the unsuccessfulness of one’s own actions.

Several interviewees described feeling sad as a reaction to the death of the simulated patient. The loss of ‘another human life’ and ambivalent attitudes towards living will were cited as causes of the grief.

Furthermore, feelings of failure, shock, anxiety, despair, disappointment, and frustration characterised the emotional perception of the situation. The disappointment and frustration referred to the lack of success of one’s actions despite the certainty about the correctness of the initiated measures.

“So I would say, so a little bit the frustration or this helplessness that that which you virtually always do, which you learn, THEN also doesn’t work. So just this/ somehow, it doesn’t work out as well as it should.” (Scenario participant).

Various interviewees described relief and contentment in response to stopping resuscitation. In particular, respecting patient’s will and witnessing that patient could get his will by stopping life-saving measures were found fulfilling.

“And I found that somehow/ actually I found it quite nice because I thought to myself, it’s really the patient’s will that is realised here.” (Medical student).

Many interviewees struggled precisely describing and naming their feelings.

Various participants described low emotional distress in response to the death of the simulated patient. The interviewees explained their low emotional involvement by the ordinariness of death in everyday clinical life – ‘it’s just part of it’. Furthermore, the extensive experience in the palliative care unit, the in-depth preoccupation with the topic of death, the attitude that death is part of life, and the circumstances of the death e.g., the absence of the relatives, were cited as causes for the low level of emotional distress.

#### Reflections on the experience

The interviewees described varying degrees of cognitive engagement with the experience. Some interviewees described that the death of the simulated patient occupied their minds even after the course, continuing to reflect on the experience for days.

“I have to say, the next two days afterwards it really occupied my mind quite heavily as well, the topic. And I noticed very clearly that it left such an impression, even though it was a played-out situation in itself.” (Scenario participant).

The death of the simulated patient evoked associations in some interviewees. The interviewees recalled resuscitations from their work practice where CPR was terminated and compared this experience with the scenario experience.

Furthermore, the questions of what could have been done differently concerning the actions in the simulation occupied our interviewees’ minds. In this regard, the interviewees addressed doubts about the correctness of the measures taken.

Several interviewees projected the situation onto themselves. The interviewees described realising how important it is to think about what one wants for oneself timely and to write down one’s own wishes. One scenario participant described speaking with the family following the scenario and addressing the importance of clearly communicating one’s own wishes regarding one’s own death within the family.

Several interviewees reported their need to talk to someone after the session. The interviewees described talking with their loved ones and colleagues about their experience. The interviewees stressed the importance of the dialogue partner being someone who has had similar experiences and can relate to the experience. At the same time, the interviewees emphasised the importance of a relationship of trust.

In this regard, the interviewees criticised the strong focus on healing and the lack of discussion of dying and death in medical and nursing education. Especially in medical studies, the coverage of the subject of death was considered insufficient.

“All the time it’s only about how we get patients to be healthy again and how/ so all this time just about that. The only time when death is addressed is during the palliative seminar, but it’s only touched upon, and apart from that it’s only about saving people all the time.” (Medical student).

Medical students complained that when death is discussed, e.g., in the context of palliative medicine, the focus is primarily placed on the well-being of the patients and the perspective of the physicians is hardly an issue. Medical students and nursing trainees agreed that their educational programs do not adequately prepare them to encounter and deal with the death of a patient. Given that each of them will one day face the death of patients, the interviewees considered it important to cover the topic of death in detail, to break the taboo of death and to learn how to deal with a patient’s death.

“And then I believe it should also be addressed normally and shouldn’t be a taboo subject. It’s terrible that death is actually still such a taboo subject.” (Nursing trainee).

Furthermore, the interviewees criticised the lack of support from medical staff after the patient’s death in the clinical setting. Lack of time was cited as a reason for the lack of reflection on and discussion of dying experiences. Consequently, interviewees called for active discussion on patients’ death and addressing the feelings of medical staff.

### Value of the experience

Our interviewees found it valuable to experience what termination of CPR and patient’s death feels like. Especially, against the context of hospital mortality rates, interviewees found it useful to practice dealing with a patient’s death in a simulation.

“Because, that is usually the day-to-day life, people die in the hospital every day, and I believe it’s also important to experience something like that for once and maybe not immediately with a patient, but first just figure it out yourself with a puppet, okay, how am I feeling in this moment.” (Nursing trainee).

Furthermore, the course experience enabled the interviewees to identify elements of good management of an emergency situation. With this regard, interviewees highlighted the moment in the scenario where the team stopped resuscitating the patient. It was criticised that the team stopped CPR too quickly without a clear approach and shared decision-making. In particular, the clear communication about the decision to stop life-saving measures was lacking.

“I found the moment where the compressions were stopped, I also said it then, that it was going a little bit too fast for me. So, I somehow would have liked for there to be clearer communication, so, or compressions would have continued, even if the advance directive was already there and a collective decision, okay, it’s fine now, we will stop, it says so here.” (Medical student).

Consequently, shared decision-making was considered by the interviewees as an important component of successful handling of an emergency situation. Communication within the team was seen as a prerequisite for shared decision-making and thus an important element for successful collaboration. In addition, the interviewees stressed the importance of a team leader taking charge, coordinating the situation and thus contributing significantly to the structured approach and successful collaboration.

Several interviewees explained that they feel better prepared for dealing with dying and death after the course. They described having a plan on how to act in such an emergency situation, knowing the next steps, what to do, and who to contact.

In general, the interviewees stated that the course experience encouraged them to reflect on dying and death, initiated discussion on the topic, and thus, increased their awareness of dying and death.

In this context, the interviewees pointed to the unrealistic nature of standard emergency training, in which simulated patients are not allowed to die. The risk of demoralisation and uncertainty were cited as reasons why patient survival is the dogma in resuscitation simulation.

“I don’t know whether they always somehow wanted to give us a feeling of success, so like, you just performed great CPR, but I find these exercises also exist for us to NOT perform great CPR for once that it DIDN’T work.” (Medical student).

However, the interviewees referred to the learning potential of such simulations and called for targeted use of this resource. One nursing trainee pointed out the impossibility of learning entirely how to deal with the dying patient in a simulated situation. Nevertheless, all participants agreed that simulation offers the opportunity to prepare oneself for such situations.

With this regard, our interviewees addressed characteristics that affect the impact of the simulation and determine its success. Three characteristics, which are described below, could be identified.

#### Unexpected character of the confrontation with death

The unexpected nature of the confrontation with the death of the patient was identified as a factor influencing the impact of the simulation. The absolute majority of the interviewees stated that they did not expect the patient to die. Several interviewees explained that they were aware of the proposition that the patient should not die in a simulation, which made the twist of the scenario all the more surprising for them.

At the same time, the interviewees appreciated the unexpected character of the encounter with the simulated patient’s death. According to the interviewees, the unexpected confrontation allowed them to experience the situation as authentically as possible and gave them the opportunity to experience what it is like when the patient dies. As ‘it came out of nowhere’, it was possible to evoke and capture the ‘unadulterated reactions’ of the participants. Our interviewees considered the unexpected character as the key to the simulation’s success.

“So, I also found it great that it somehow, that the simulation, that it happened like that and that also nobody knew about it beforehand, because, through this shock, everyone was somehow forced to, like everyone was drawn out of their shell a little bit, to somehow express themselves and also to face it.” (Scenario participant).

#### The advanced directive

The importance of the AD was repeatedly highlighted by our interviewees. The interviewees valued the opportunity that the higher authority in the form of the patient’s will decided on the outcome of the resuscitation and that they did not have to make the difficult decision themselves to let the patient die. Thus, several interviewees perceived the appearance of living will as a clarifying event that relieved them of the decision-making. The AD was considered as final note that determined the further course of events and thus resolved uncertainty and was seen as a relief. Furthermore, the interviewees found relief in knowing that it was not the team’s medical malpractice that led to the patient’s death. Consequently, the AD prevented them from interpreting the patient’s death as their own failure. At the same time, the AD made it easier for the interviewees to deal with the outcome. One scenario participant indicated that to know that it was the patient’s wish to forgo life-saving measures made it easier for them to accept the outcome of the scenario.

“I would also have found it more tragic if the scenario would have been aborted, before you would have succeeded and the patient would have simply died, but it was alright with the advance directive in mind, so it was reasonable.” (Scenario participant).

#### Debriefing

The debriefing was identified as third factor that determines the learning success of the simulation. Interviewees agreed that debriefing intensified the impact of the experience. Our participants reported greater emotional involvement as a result of reflection during debriefing.

“The more we talked about it, the more I was actually affected by it.” (Medical student).

Interviewees agreed that talking and reflecting on the experience enabled them to consciously perceive and process their own feelings. Observing that other participants were similarly affected by the experience was seen as comforting.

In this regard, the role of the trainers in debriefing, who led the discussion, was highlighted. The interviewees particularly valued the expertise of the trainers and their rich experience with difficult situations, termination of resuscitation and patient death.

“I found the discussion afterwards very, very good because there were also people there, who experience something like that more often, and obviously they would talk with you about it, […] that wasn’t so bad at all, that it’s not some random, bored Internist who runs this course, but somebody who of course has a certain motive and who also has experience.” (Scenario participant).

Interviewees valued the open and honest approach of the trainers. They appreciated hearing about trainers’ personal experiences with patients’ loss and learning from experienced professionals that patients’ death still affects them emotionally. Allowing and actively addressing feelings by the trainers during the debriefing encouraged participants to allow and perceive their own feelings as well. Observing trainers’ emotional distress led the interviewees to realise that it is okay to develop strong feelings in response to the death of a patient, that it is ‘only human’ to be concerned about a patients’ death and to admit feelings.

“I somehow found it very valuable to hear it like that for once, that now when the patient dies, that it’s okay and that it’s also important that that kind of situation stays on one’s mind, and that one also feels bad or terrible with a situation like that.” (Nursing trainee).

Furthermore, interviewees found it useful to learn how professionals deal with patient death, what coping strategies they use and that everyone has his/her own strategy to deal with a patient’s death. Interviewees recognised that it is good to talk to others about the patient’s death and even to actively seek exchange. In this context, interviewees emphasised the importance of support from and exchange with team colleagues. According to our interviewees, it was valuable to learn that there are opportunities and that opportunities should be created to discuss these issues.

“That it’s also important to discuss the whole thing at that point and that there are also opportunities or that opportunities are created to talk about these subjects. I would definitely draw a lesson from that and would try to keep this in mind, that it’s okay to talk about it.” (Nursing trainee).

Hearing from experienced anaesthesiologists and intensive care nurses that it takes time to process the experience gave interviewees confidence for dealing with a patient’s death in the future. It was also positively highlighted that the team of trainers was interprofessional.

Various interviewees positively highlighted that sufficient time was taken for debriefing. According to the interviewees, the duration of the debriefing of approximately two hours allowed for a detailed and in-depth exchange. Furthermore, the individual approach was emphasised. The fact that each course participant was given the opportunity to express themselves and that each person was dealt with individually was appreciated. The practical character of the debriefing and the practical suggestions were also valued. In this context, the question of when to stop resuscitating patients and let them die was stressed. The interviewees appreciated the opportunity to record the scenario and watch it later, and thus the opportunity to look at it again from the outside to and ask themself how they could have reacted better in the situation. Several interviewees stated to have found the debriefing even more valuable than the simulation itself.

“This whole exchange afterwards, I found it significantly more valuable than the actual Simulation.” (Scenario participant).

The interviewees also appreciated the offer to the participants to talk about the experience beyond the course.

## Discussion

The present study was designed to investigate how medical students and nursing trainees perceive and experience the sudden death of a patient in simulation training. We strived to explore how and under what conditions it can be valuable to simulate the death of a patient. Therefore, we developed an interprofessional, simulation-based course with one session focusing on termination of resuscitation and subsequent debriefing.

### Perception and experiences of medical students and nursing trainees

We could observe that simulated death caused varying degrees of emotional distress in the participants. The intensity of the consternation was attributed to the novelty of the experience. Negative feelings, such as shock, uncertainty, distress, disappointment, sadness, anxiety, failure, despair, doubt and frustration dominated the emotional perception of the experience. Other authors described in the review of Ho et al. observed similar outcomes; students often reported negative emotional outcomes, e.g., shock, confusion, emotionally overwhelmed or inability to find the “right words”. A minority describes physical reactions such as throat tightness or paraesthesia [5]. In other simulations, students also reported positive effects, such as memorable, powerful, inspiring, or transformative [5]. These effects were not reported by our interviewees. However, our results illustrate the importance of actively forgoing life-sustaining measures despite available treatment options, which was perceived as particularly formative by our participants and evoked helplessness in them. In addition to the negative feelings, various interviewees described relief and contentment in response to stopping resuscitation. Especially, respecting patient’s wishes by stopping life-saving measures were perceived fulfilling. Thus, respecting the patient’s wishes can be assumed to be one central medical task in dealing with a dying patient.

Our interviewees described varying degrees of cognitive engagement with the experience. The simulation evoked associations in some participants. The questions of what and how things could have been done differently concerned them. Various participants projected the situation onto themselves and described having realised how important it is to think in time about what one wants for oneself and to put one’s own wishes in writing. Moreover, the interviewees reported that they felt the need to talk to someone about their experiences and stressed the importance of the dialogue partner being someone who has had similar experiences and can relate to it. Similar reactions were described by the medical students who were involved in the care of dying patients [5].

### Value of death simulation

Similar to Wynter and Brignal [2], our participants criticised the strong focus on healing and the lack of discussion of dying and death in medical and nursing education. In addition, Wynter and Brignal describe that medical and nursing students feel most unprepared for this part of their future job and are worried about being responsible for negative patient experiences due to their unpreparedness [2]. The results of the cross-sectional study of Ioshimoto et al. show that medical students with more training in end-of-life care have more interest in the topic, are better prepared to care for dying patients, and have more experience in caring for terminal patients [30]. Our interviewees missed adequate preparation for dealing with the death of patients during their studies and they felt let down by the education system. The medical students complained that when death is discussed, the focus is primarily placed on the well-being of the patients and the perspective of the physicians is hardly an issue. The participants’ reaction to the living will that they had been lured into a trap illustrates these shortcomings. The death of the simulated patient was perceived as an ambush, as this was not the case in previous teaching sessions and all patients always survived in the context of emergency situations. This critique highlights the importance and necessity of developing teaching approaches that foreground students’ subjectivity and perspective in the context of palliative care education and beyond. Here we are in the core of the project to experience exactly this ambush in the protected environment and in the following to get possible coping strategies and thus security with such situations.

Moreover, interviewees criticised the lack of support from medical staff after the death of a patient and addresses scarce time resources as reason for the lack of reflection. Precisely the emotional and professional support from ward staff is identified as an important source for medical students to learn from patient death [31]. Thereby, the support should be structured (e.g. periodic group meetings), active (e.g. check if students are okay even if they do not ask for help), sensitive (e.g. non-judgemental), and include peers and near-peers [31]. Students of a similar course examined by Mileder et al. found the availability of psychological support after death simulation particularly valuable [32]. We would therefore like to encourage medical schools to include the topic of death more strongly in medical and nursing education and to promote support and exchange in both training sessions and clinical practice. Our interviewees reported that they felt the need to share their common experience and stressed the importance of the dialogue partner being someone who has had similar experiences and can relate to the experience. This coping strategy is also described by students in the study by Trivate et al.; here, students reach out to peers and near-peers, consultants, or family members to discuss their experience and turn it into a lesson learned for their professional life and role [31].

Medical students and nursing trainees found the confrontation with death in the simulation setting valuable in many ways. On one hand, the simulation experience allowed our interviewees to realise their knowledge gaps in dealing with emergency situations. On the other hand, the course provided the practical knowledge and skills needed to deal effectively with emergency situations. On a more general level, the simulation experience encouraged our participants to reflect on dying and death, initiated discussion on the topic, and thus, raised their awareness on the topic. Overall, our participants appreciated the opportunity to experience what it feels like to lose a patient and after the simulation they feel better prepared to face dying and death. We share this observation with Ho et al. who found similar experiences of students facing death, e.g. they felt more comfortable, confident, and prepared for managing their dying patients [5].

### Conditions of simulation training

Bruppacher et al. described simulation training as a tool to increase emotional tension and stress to improve students’ clinical practice using fear-based-motivation. Although they have some concerns about the simulation of death, they no doubt agree that simulation education is a useful training-tool [9]. In our interviews, students referred to the learning potential of confronting death in simulation setting and called for a targeted use of this resource. With this regard, we could identify several characteristics that influence the perception of the experience and determine the impact of the simulation: the unexpected confrontation with the patient death, the AD and the debriefing. The unexpected confrontation allowed our participants to experience the situation as authentically as possible and gave them the opportunity to experience what it is like when a patient dies. Evidence shows a direct relation of participants who experienced simulated unexpected death and a better performance on further assessment scenarios [33]. Therefore, we encourage schools to offer the unexpected death simulation training in other courses and to make it a regular part of the curriculum, despite the risk of emotional overload for learners, as discussed in the literature [3].

Furthermore, our study highlights the impact of the AD on the perception of the simulation. Our interviewees described strong feelings as reaction to the appearance of the AD. The fact that a higher authority in form of an AD decided the outcome of the situation and that the participants did not have to make the difficult decision themselves to let the patient die was perceived as relieving. In addition, the certainty that it was not due to the poor performance of the team that the patient died provided comfort. Further, knowing that it was the patient’s wish to forgo life-saving measures made it easier for the interviewees to accept the outcome of the scenario. On a more general level, our study illustrates the burdensome nature of the AD and the difficulties medical students and nursing trainees have in dealing with it. The uncertainty about the circumstances under which the patient’s will was drafted and whether the patient was able to fully comprehend the meaning of the living will leads to an ambivalent attitude of the interviewees towards the AD. Even physicians feel uncomfortable dealing with AD, but they agree that AD are fundamental for the decision to stop resuscitation [16].

Moreover, our study highlights the importance of debriefing. Debriefing can positively impact the psychological outcomes of healthcare providers experiencing a patient’s death [34]. Thereby, the types of debriefing models differ; while in simulation situations the focus is mostly on learning, in clinical settings the focus is on emotional reactions [34]. In general, in either environment is a need for debriefing [34]. In our debriefing the focus was on talking about and reflecting on the experience. This enabled our interviewees to consciously perceive and process their own feelings and led to greater emotional involvement. In this context, the role of the trainers was highlighted, especially their rich experience with resuscitations and death of patients. Moreover, the open and honest approach of the trainers is emphasised. Allowing and actively addressing feelings by the trainers during the debriefing encouraged participants to allow and perceive their own feelings as well. Additionally, the interprofessional composition of the team of trainers, sufficient time, the practical character, the individual approach and the possibility to record the scenario and view it later are identified as factors that determined the impact of the course.

Overall, our study illustrates the didactic value of confronting death in simulation settings. It provides insights into medical students’ and nursing trainees’ perceptions and offers guidance on the challenges associated with simulating the patient’s death. Moreover, our study demonstrates that simulation training with the subsequent debriefing can be an effective way to sensitise trainee health professionals to death and better prepare them for encounters with dying patients.

### Limitations of the study

Our study has some limitations. The findings are based on a one-time simulation event which is hard to generalise from. Apart from the single-center aspect, due to the qualitative methodological approach, extrapolating the findings of this study to other contexts may be difficult. With respect to the medical students, there might have been a selection bias, since their participation was on a voluntary basis. This may have resulted in the participating medical students being more motivated and interested in emergency case management and simulation-based training. Furthermore, the bias of the moderators, which can affect the outcome of the focus group, should be noticed. We attempted to mitigate this risk by close prior consultation of the moderators, detailed discussion of the focus group objectives, and the use of a semi-structured interview guide. Nevertheless, there is a risk that the general sense of the subject and differences between groups that an individual interviewer would get was compromised. We also cannot rule out the possibility that the sensitivity of the topic or possible peer pressure or hierarchical structures influenced the conversations in the focus groups and thus biased the data. The time interval of one week between the simulation experience and the focus groups should also be addressed as a limitation, as there is a risk that memories will be subjectively adjusted over time. However, the time interval allowed us to capture ‘long-term’ reflections on the experience beyond spontaneous reactions. Furthermore, the realistic nature of the simulation should be questioned. Our participants had no prior knowledge about the patient. It can also be assumed that the external appearance of the patient – a high-fidelity simulator dressed in patient clothing – affected the realistic character of the simulation. However, it can be argued that similarities to the real world promoted transfer. Nevertheless, our study provides rich insights into medical students’ and nursing trainees’ perceptions of the sudden death of a simulated patient and enriches knowledge about the factors that determine the success of simulation training.

## Conclusions

Our study shows that confronting with death in simulation training induces different degrees of emotional distress and cognitive engagement in medical students and nursing trainees, and that negative feelings dominate the emotional perception of the experience. Further, our results illustrate the deficits in dealing with the topic of death in medical and nursing education by discussing the lack of adequate preparation for dealing with death. At the same time, our results underline simulation training as an effective tool to prepare health workers for encounters with dying patients and to increase their awareness of the topic. Our study shows that the unexpected nature of the confrontation, the presence of the advance directive, and debriefing are critical to the impact of the simulation and thus provides practical guidance for designing such interventions.

### Electronic supplementary material

Below is the link to the electronic supplementary material.

Supplementary Material 1

## Data Availability

The datasets generated and/or analysed during the current study are not publicly available due the organisation’s policy but are available from the corresponding author on reasonable request.

## Footnotes

1 All quotes have been translated verbatim.

## Declarations

### Guidelines

All methods were carried out in accordance with Declaration of Helsinki.

## Abbreviations

AD: Advanced Directive
GT: Grounded Theory
TUM: Technical University of Munich
