# Supplementary material for: Experiences of medical students and nursing trainees from unexpected death through simulation training
Source: BMC Med Educ. 2023 Sep 14;23:667. doi: 10.1186/s12909-023-04638-x (PMC10503193; doi:10.1186/s12909-023-04638-x)
Supplement: Supplementary file 1 — Supplementary Material 1 [file 12909_2023_4638_MOESM1_ESM.docx]

**Additional file 1**

**Interview guide for the focus groups**

1. Can you remember the last session when the patient died in the simulation? What exactly happened there?
2. How was it for you? What were you thinking? What did you feel?
3. Were there any special moments in the scenario?
   1. … particularly challenging/difficult moments?
   2. … beautiful/fulfilling moments?
   3. Were there any special moments in the teamwork?
4. Did the experience still bother you after the course? In what situation?
   1. What thoughts did the simulation experience trigger in you?
   2. What feelings did dealing with the experience evoke in you?
5. Was this simulation experience valuable for you? In what way?
6. How do you evaluate the handling of the topic in the session? The idea of the simulation patient dying and its implementation?
7. Is there anything else you would like to share?
